# The Quality of Life Impact Refractive Correction (QIRC) questionnaire: validation of the Malay-translated version of the QIRC using Rasch analysis

**DOI:** 10.1186/s12886-021-02145-5

**Published:** 2021-10-25

**Authors:** Md Mustafa Md-Muziman-Syah, Nur Solehah Muzir, Haliza Abdul Mutalib, Noorhazayti Ab. Halim

**Affiliations:** 1 Department of Optometry and Visual Science, Kulliyyah of Allied Health Sciences, International Islamic University Malaysia, 25200 Kuantan, Pahang Malaysia; 2 Centre for Community Health Studies, Program of Optometry and Visual Sciences, Faculty of Health Sciences, Universiti Kebangsaan Malaysia, Jalan Raja Muda Abdul Aziz, 50300 Kuala Lumpur, Malaysia; 3 Department of Public Health, Kulliyyah of Dentistry, International Islamic University Malaysia, 25200 Kuantan, Pahang Malaysia

**Keywords:** Validation, Translation, Cross-cultural adaptation, QIRC, Malay version, Malaysian population, Rasch analysis, Quality of life, Refractive correction

## Abstract

**Background:**

The Quality of Life Impact Refractive Correction (QIRC) questionnaire is a Rasch-validated instrument to assess the quality of life of ametropes with refractive correction. The original QIRC was validated in the United Kingdom. This study aimed to validate the Malay version of the QIRC among refractive correction wearers in Malaysia using Rasch analysis.

**Methods:**

The original 20-item QIRC was forward-backward translated into Malay in preparation for the Pilot Malay QIRC. The pilot version was pre-tested on 105 spectacle/contact lens-corrected myopes, and the results were reviewed and cross-culturally adapted to produce the Final Malay QIRC. The final version was self-administered to a new sample of 304 participants. A Rasch analysis was conducted to evaluate the items and response categories of the Pilot and the Final Malay QIRC. Test-retest reliability was also analysed on the Final Malay QIRC.

**Results:**

Based on the pre-test findings, Rasch analysis revealed a multidimensional scale (functional scale [Items 1 to 13] and emotional scale [Items 14 to 20], which were separated in subsequent analysis), unordered response categories for the functional scale (Category 3 was collapsed into Category 2), one misfit item (Item 3 was removed) and six items required modification (Items 4, 6 to 9, and 12 were reworded and cross-culturally adapted). In the Final Malay QIRC, both the functional and emotional scales had ordered response categories, good person reliability (functional, 0.80; emotional, 0.81) and separation index (functional, 2.01; emotional, 2.06), well-targeted items (targeting precision: functional, 0.28 logits; emotional, 0.08 logits), and satisfactory fit statistics (infit and outfit mean square were less than 1.50 for all items). A noticeable differential item functioning (DIF) between genders was found in Item 18 (DIF contrast, 0.40 logits; *p* = 0.04). Test-retest reliability analysis demonstrated a high intraclass correlation coefficient (0.94) and Cronbach’s alpha (0.97) with a coefficient of repeatability of ±8.14 units.

**Conclusions:**

The Malay-translated version of the QIRC has good psychometric characteristics for assessing the quality of life of refractive correction wearers in Malaysia. This translated and cross-culturally adapted Malay QIRC is a valid and reliable instrument that can be used in routine clinical practice.

## Introduction

Uncorrected refractive error is the leading cause of low vision in Malaysia and even worldwide [1, 2]. Several methods are available to correct refractive error, either by wearing spectacles or contact lenses or by undergoing laser refractive surgery. All refractive correction methods aim to restore clear vision, which concomitantly improves the quality of life (QoL) of ametropes. Visual acuity is a commonly measured clinical parameter to assess the improvement of visual function. The information derived from this objective clinical measure is subtle to represent the overall visual function improvement after the correction given. Patient self-evaluated QoL provides the patient’s perspective on visual function improvement with the refractive correction to complement the objective clinical measure. The combination of the objective and subjective measures offers a holistic assessment to reflect the overall improvement in visual function and patient’s satisfaction.

A previous review reported that questionnaires developed and validated using Rasch analysis have superior psychometric properties [3]. Rasch analysis converts ordinal scores from response categories into logits, which are equal-interval measure units. In addition, the analysis can evaluate both participant ability and item difficulty [4]. Therefore, it can be used to determine whether a developed questionnaire is well-targeted to or deviated from the intended group. There are three Rasch-validated questionnaires available to assess the impact of refractive correction on vision-related QoL [3], namely the Quality of Vision (QoV) [5], the Near Activity Visual Questionnaire (NAVQ) [6], and the Quality of Life Impact on Refractive Correction (QIRC) [7]. The QoV mainly assesses patients’ visual symptoms such as glare and halos after refractive surgery, while the NAVQ specifically evaluates the activity limitations in patients with presbyopic correction. The QIRC covers comprehensive aspects of QoL, including functional and emotional in patients with refractive correction.

The QIRC was developed and validated in the English-speaking population of the United Kingdom [7]. However, this questionnaire is also available in Spanish, Greek, Dutch [8] and Chichewa [9]. Malaysia is known as a multi-ethnic country in which Malay is the largest population (69.6%), followed by Chinese (22.6%), Indian (6.8%), and others (1%) [10]. Furthermore, the Malay language is gazetted as the national language of Malaysia [11]. Thus, the Malay version of the QIRC is essential for assessing the QoL of Malaysians with refractive correction.

Each refractive correction method serves a different impact on patients’ QoL [12, 13]. Spectacles and contact lenses have become a primary mode of correction over the past few decades [14]. Spectacle correction is a cost-effective option [15] because it can be worn full-time, requires low maintenance, and has a low risk of complications. Contact lens correction has gained popularity among teenagers [16] as it is relatively inexpensive and easily accessible in Malaysia [17]. In contrast, patients must meet preoperative criteria for refractive surgery mode, and the procedure involves additional costs for postoperative follow-up. Hence, this study aimed to translate the original QIRC into Malay and validate the translated version using Rasch analysis on spectacle/contact lens-corrected myopes in Malaysia.

## Methods

The validation of the Malay-translated version of the QIRC involved two stages: the Pilot and the Final Malay QIRC versions. Both stages underwent linguistic and/or psychometric validations. The linguistic validation consisted of forward-backward translation, translation discrepancy and consistency checking, a pre-test of the Pilot Malay QIRC and a review of ambiguous items reported in the pre-test. The psychometric validation of the Pilot and the Final Malay QIRC was evaluated using Rasch analysis. Additional test-retest reliability analysis was conducted on the Final Malay QIRC.

### The QIRC questionnaire and the translation

The QIRC was designed to assess the impact of refractive correction on QoL either through spectacles, contact lenses or refractive surgery. It consists of five domains; visual function (Item 1), visual symptom (Item 2), visual convenience (Items 3 to 7), visual concern (Items 8 to 13), and emotional well-being (Items 14 to 20) [7]. Five response categories were used to score each item of visual function and visual convenience (Category 5 = *extremely* to Category 1 = *not at all)* and the other five response categories for each item of visual symptom, visual concern, and emotional (Category 5 = *always* to Category 1 = *never).* One additional response category (Category 0 = *don’t know/not applicable)* was included for all items and was considered as missing data [18].

The Malay QIRC went through the standard protocol for forward-backward translation. First, a professional translator (AAA) and an optometrist (NSS), who were Malay-English bilinguals, independently translated the original QIRC into Malay. A panel of experts analysed the content equivalence of the translation with the original version [19] and the suitability of the translated phrases to the culture of the target population. The experts then resolved the discrepancies between the two Malay translations [20]. The panel consisted of two experts in the QIRC measures (MMMMS, HAM), an expert in the questionnaire validation process (NAH) and an expert teacher of the Malay language (AHJ), and all were proficient in the Malay-English languages.

The other two blinded bilingual Malay-English translators, an optometrist (AFF) and a family medicine specialist (MSES), translated the Malay version back into English. Three of the researchers (MMMMS, HAM, NAH) assessed the consistency of the backward translations with the original QIRC and achieved a consensus translation of the Pilot Malay QIRC.

The pre-test was conducted by one researcher (NSM) on 105 participants who were bilingual Malay-English speakers. The participants self-administered the 20-item Pilot Malay QIRC and commented on any ambiguous items. The time taken to administer the questionnaire was recorded (mean, 9.76 ± 0.66 min). Subsequently, the pre-test output was reviewed by all researchers (MMMMS, HAM, NAH, NSM) to enhance the item comprehension and cross-cultural adaptation.

### Participants

This cross-sectional study was conducted at an institutional optometry clinic in Kuantan, Pahang, Malaysia, and at a general private optometry clinic in Gombak, Kuala Lumpur, Malaysia, from 4 February 2019 to 28 February 2020. Participants were randomly selected from patients attending their optometric appointment using Research Randomizer version 4.0 [21, 22]. The Malay QIRC was self-administered by non-presbyopic participants who were able to understand the Malay language. Only participants with refractive error of − 3.00 D and above in spherical equivalent refraction (SER) and astigmatism correction of less than 2.00 D were included. All participants wore their current spectacle or contact lens correction prescribed by optometrists for less than 1 year. Patients with ocular or systemic disease, ophthalmic surgical history, or trauma noted in the previous medical record or optometric examination were excluded from this study.

This study design was granted approval by the International Islamic University Malaysia Research Ethics Committee (IIUM/504/14/11/2/IREC2019-KAHS[U]). Written consent was gained from all eligible participants. The study protocols were operated in compliance with the tenets of the Declaration of Helsinki, the International Conference of Harmonisation Good Clinical Practice Guideline (ICH-GCP) and the Council for International Organisations of Medical Sciences (CIOMS) International Ethical Guidelines.

### Sample size

Sample size determination was calculated for the 20-item QIRC with five response categories at a power of 0.80 and a probability level of 0.05 using Free Statistics Calculators version 4.0: A Priori Sample Size for Structural Equation Models [23]. The minimum sample size required for the model structure was 100. The suggested sample size was adequate for Rasch analysis based on a 95% confidence of item calibrations or person measures stable within ±0.5 logits [24]. Furthermore, it corroborated the guidelines for respondent-item ratios, ranging from 4:1 to 15:1 [25, 26].

### Statistical analysis

Psychometric validation of the items and response categories was performed using Rasch analysis and test-retest reliability analysis. Rasch analysis was employed using the Andrich rating scale model (Winsteps software version 4.5.1). While test-retest reliability analysis was conducted using Statistical Package for the Social Sciences (SPSS) software for Windows version 25.0.

### Pilot Malay QIRC

First, Rasch principal components analysis of residuals (PCAR) was employed to identify the unidimensionality of the scale. Unidimensionality is crucial as it demonstrates that the instrument measures the underlying trait for which it was designed [27]. In this context, PCAR is used to determine if the residuals exhibit patterns after accounting for the observed variance explained by the Rasch measure. PCAR examines contrasts in the correlation matrix of the residuals [28]. The first contrast refers to the component that accounts for the greatest amount of variance in the residuals. The Rasch (first) dimension should achieve a variance explained by measures of 50% [28]. Unexplained variance in the first contrast of 3.0 Eigenvalue units or greater indicates the presence of an additional (secondary) dimension that is not considered random noise [28]. The PCAR result revealed multidimensionality in the Malay QIRC version. The variance explained by measures was less than 50%, with unexplained variance in the first contrast being more than 3.0 Eigenvalue units. Hence, the Malay QIRC was divided into the functional (Items 1 to 13) and emotional (Items 14 to 20) scales, and subsequent analyses were conducted separately.

**Fig. 1:**
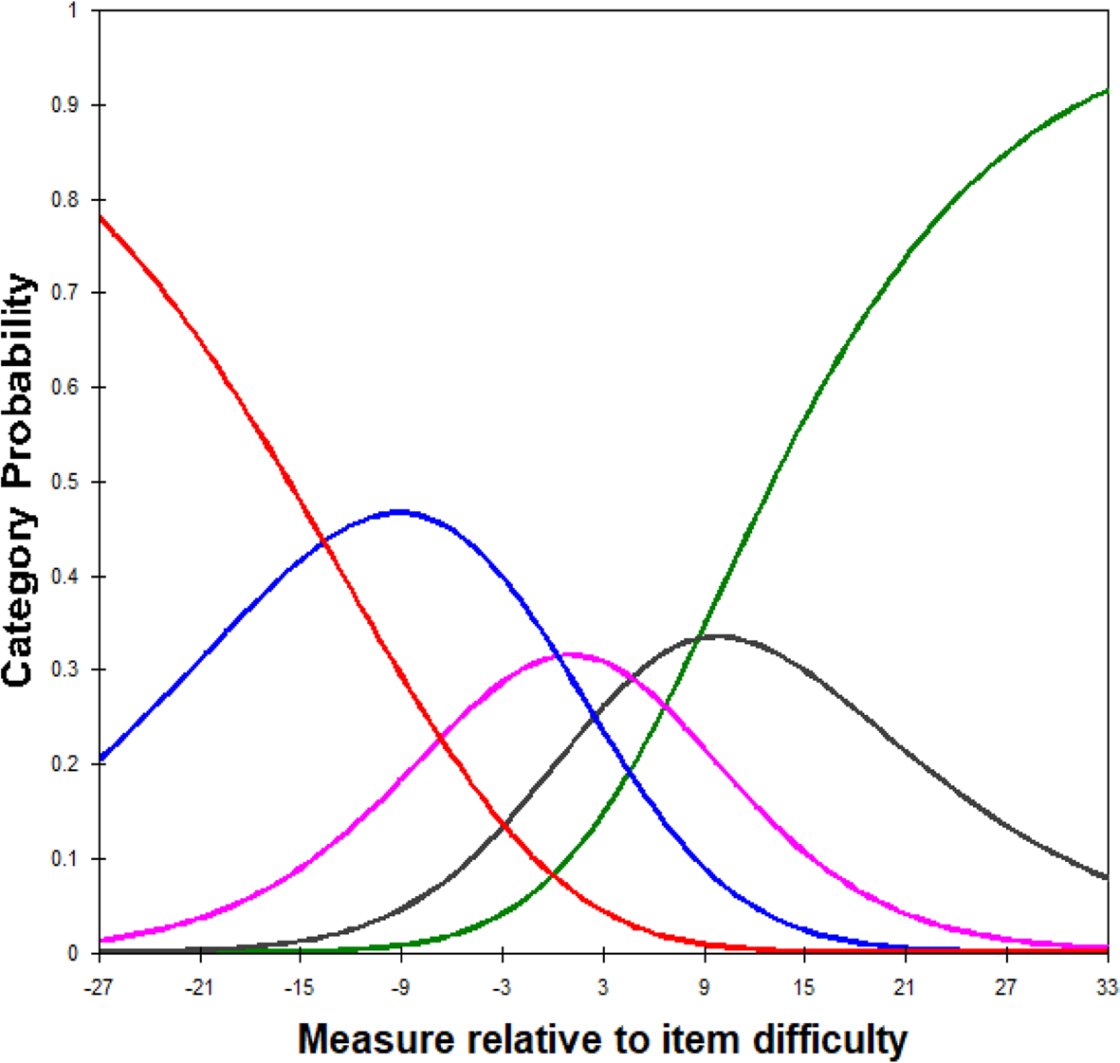
Category probability curve for the functional scale of the Pilot Malay QIRC. Red = Category 1 (*not at all/never*); Blue = Category 2 (*little/occasionally*); Pink = Category 3 (*moderate/fairly often*); Black = Category 4*,* (*a lot/very often*); Green = Category 5 (*always/extremely*)

Next, the category probability curve was analysed to determine the order of response categories. The category probability curves show that the response categories for the functional scale were unordered (Fig. 1), while the response categories for the emotional scale were ordered. Thus, the unordered category for the functional scale was collapsed into an adjacent category. The new four response categories for the functional scale were applied in the Final Malay QIRC.

Then, the items fit were analysed to determine any misfits. The following item removal criteria were set: Items infit and outfit were outside 0.50 to 1.50 mean square (MnSq) or were equal to/greater than 2.0 z-standardised (z-std) [28], missing data were greater than 50%, floor and ceiling effects were higher than 50%, and skewness and kurtosis were out of − 2.00 to + 2.00 [7]. Analysis of the item fit statistics identified one misfit item, indicating removal of the item. As a result, a version called Final Malay QIRC was prepared with 19 items.

### Final Malay QIRC

A Rasch analysis was performed to evaluate the psychometric properties of the Final Malay QIRC, consisting of 12-item functional and 7-item emotional scales. The validity and reliability of the psychometric properties were analysed using category threshold, separation indices, fit statistics, targeting precision, differential item functioning (DIF), and test-retest reliability. A new sample of 304 participants was recruited for the psychometric validation of the Final Malay QIRC. The time spent on the questionnaire was documented (mean, 8.22 ± 0.41 min).

The category threshold was examined to assess the functionality of the response categories. The modified four response categories (after collapsing) for the functional scale were evaluated as to whether they were ordered or needed further modification. The original five response categories for the emotional scale were also re-evaluated.

The person reliability and separation index were used to evaluate the capability of the 19-item Malay QIRC to distinguish the participants’ strata. The coefficient of person reliability ranges from 0 to 1 designates the item measures precision. A higher coefficient represents a better performance of the item measures in distinguishing participant ability between different strata. In this study, a minimum of 0.80 and 2.00 were set for the person reliability and separation index, respectively [28].

The overall fit of the Final Malay QIRC to the Rasch model was analysed using the fit statistics. In this study, the researchers set the criteria for the overall fit to be 0.50 to 1.50 MnSq or less than 2.0 z-std of the average infit and outfit. Therefore, the fit statistics determined whether the Final Malay QIRC has a good fit to the Rasch model [28].

Targeting precision was determined by analysing the distribution of item difficulty to participant ability in the person-item map. The difference between the means of the person and item measures (mean difference) should be lower than 0.50 logits to signify that the QIRC items were properly targeted to the refractive correction group [29]. The person-item map also illustrates the item difficulty order for refractive correction wearers, from easiest to most difficult.

DIF was evaluated between two subgroups of participants classified by gender (male versus female) and SER (moderate myopia: ≤ − 3.00 D to > − 6.00 D versus high myopia: ≤ − 6.00 D). A noticeable DIF was considered present if the DIF contrast was greater than 0.50 logits and the probability was meaningful (*p* < 0.05) [28].

Test-retest reliability was conducted on 59 participants (out of 304 participants) during their optometric follow-up appointments at an interval of 2 to 4 weeks (median, 2.7 weeks; mean, 2.7 ± 0.6 weeks). The interval was sufficient to mask the participants’ memory on the construct being measured and its stability over time [30, 31]. In addition, the time spent completing the questionnaires at the first (mean, 8.20 ± 0.39 min) and second visits (mean, 8.08 ± 0.53 min) was tracked. Test-retest reliability analysis included the intraclass coefficient (ICC), Cronbach’s alpha (α) and coefficient of repeatability (CoR).

The ICC of greater than 0.90 reflects excellent reliability for clinical purposes. The ICC test of the two-way mixed model and consistency type was performed as described elsewhere [32, 33]. Cronbach’s α is a reliability coefficient used to measure a set of items that are consistently related as one group. It was set at 0.90 to consider that the questionnaire items had excellent internal consistency. The CoR is a standard deviation of the test-retest difference multiplied by two. Thus, a smaller CoR indicates higher repeatability.

## Results

### Pilot Malay QIRC

One hundred and twenty-three eligible participants were randomly selected to take part in the pre-test. The response rate was 85.4%, with 18 participants unwilling to participate. Thus, a total of 105 participants self-administered the Pilot Malay QIRC. Majority of the participants were Malays who came from various work statuses. Most of them were spectacle wearers, with a current correction at around 6 months of age, and had more than 10 years of experience wearing corrections (Table 1).

**Table 1:** Demographic data of the participants

Parameters	20-Item Pilot (***N*** = 105)		19-Item Final (***N*** = 304)	
	*n*	%	*n*	%
Age (year)				
Mean (SD)	26.5 (5.2)		26.2 (5.5)	
Range	18 to 38		18 to 39	
Gender				
Male	32	31	71	23
Female	73	69	233	77
Race				
Malay	88	83.8	277	91.1
Chinese	12	11.4	18	6.0
Indian	5	4.8	8	2.6
Others	–	–	1	0.3
Work status				
Government-employed	17	16.2	76	24.9
Private-employed	13	12.4	80	26.3
Student	58	55.2	102	33.5
Self-employed	13	12.4	19	6.2
Unemployed	4	3.8	27	9.1
Correction type				
Spectacle	71	67.6	198	65.1
Contact lens	10	9.5	33	10.9
Combination	24	22.9	73	24.0
Correction history (months)				
Age of current correction, mean (SD)	6.3 (3.4)		7.7 (2.5)	
Wearing experience, mean (SD)	158.2 (36)		142.7 (41.2)	

*N* number of participants, *n* number, *%* percentage, *SD* standard deviation

Based on the pre-test feedback, two items had to be modified to improve comprehension and adaptation to the local culture. Item 6 *‘swimming in the sea’* was reworded to *‘swimming at the beach’,* and Item 7 *‘when using a gym/doing keep-fit classes/circuit training’* was paraphrased to *‘during exercise or sports activities’.* Items 4, 8, 9, and 12 were amended to include *‘LASIK’* as an example of *‘refractive surgery’* to enhance the ability of the items to be self-administered.

The unidimensionality of the QIRC was investigated prior to further analysis of the validity and reliability of its psychometric properties. PCAR showed that the variance explained by the measures and the unexplained variance in the first contrast were 48.7% and 5.1 Eigenvalue units (13.1%), respectively. Further analysis of the standardised residual loadings revealed that all emotional items (Items 14 to 20) had residual loadings of greater than 0.60. These findings indicated that the Malay QIRC was a multidimensional scale. It should therefore be considered separately. It also highlighted the critical nature of Rasch analysis in translation versions, even when the original instrument was developed and validated using Rasch analysis.

As illustrated in the category probability curve, the five response categories for the 13-item functional scale were unordered (Fig. 1). The sunken category (Category 3 *= a moderate amount/fairly often*) was collapsed into the adjacent category (Category 2 *= a little bit/occasionally*). The modified four response categories were later used for the functional scale of the Final Malay QIRC.

The item fit statistics showed that infit and outfit MnSq (z-std) for Item 3 were 1.69 (4.2) and 1.78 (4.5), respectively. It reflected that Item 3 did not fit the Rasch model when MnSq was above 1.50 and z-std was greater than 2.0. In contrast, the other items fulfilled the criteria for item fit. Therefore, Item 3 was removed, and the following items were re-numbered one step up in sequence. For instance, Item 4 was re-numbered as Item 3, and the next items followed. The 19-item Final Malay QIRC was set for the further validation process.

### Final Malay QIRC

Out of 366 eligible participants who were randomly selected, a total of 304 participants (response rate, 83.1%) agreed to be recruited for the psychometric validation of the Final Malay QIRC. Participants were age-matched to the pre-test group (mean difference, 0.26 years; *t* = 0.42, *p* = 0.67). Most participants were Malays from various work statuses with more than 10 years of experience wearing corrections (Table 1). The time spent administering the questionnaire was significantly reduced for the Final Malay QIRC compared to the pilot version (mean difference, 1.53 min; *t* = 22.2, *p* < 0.001).

**Fig. 2:**
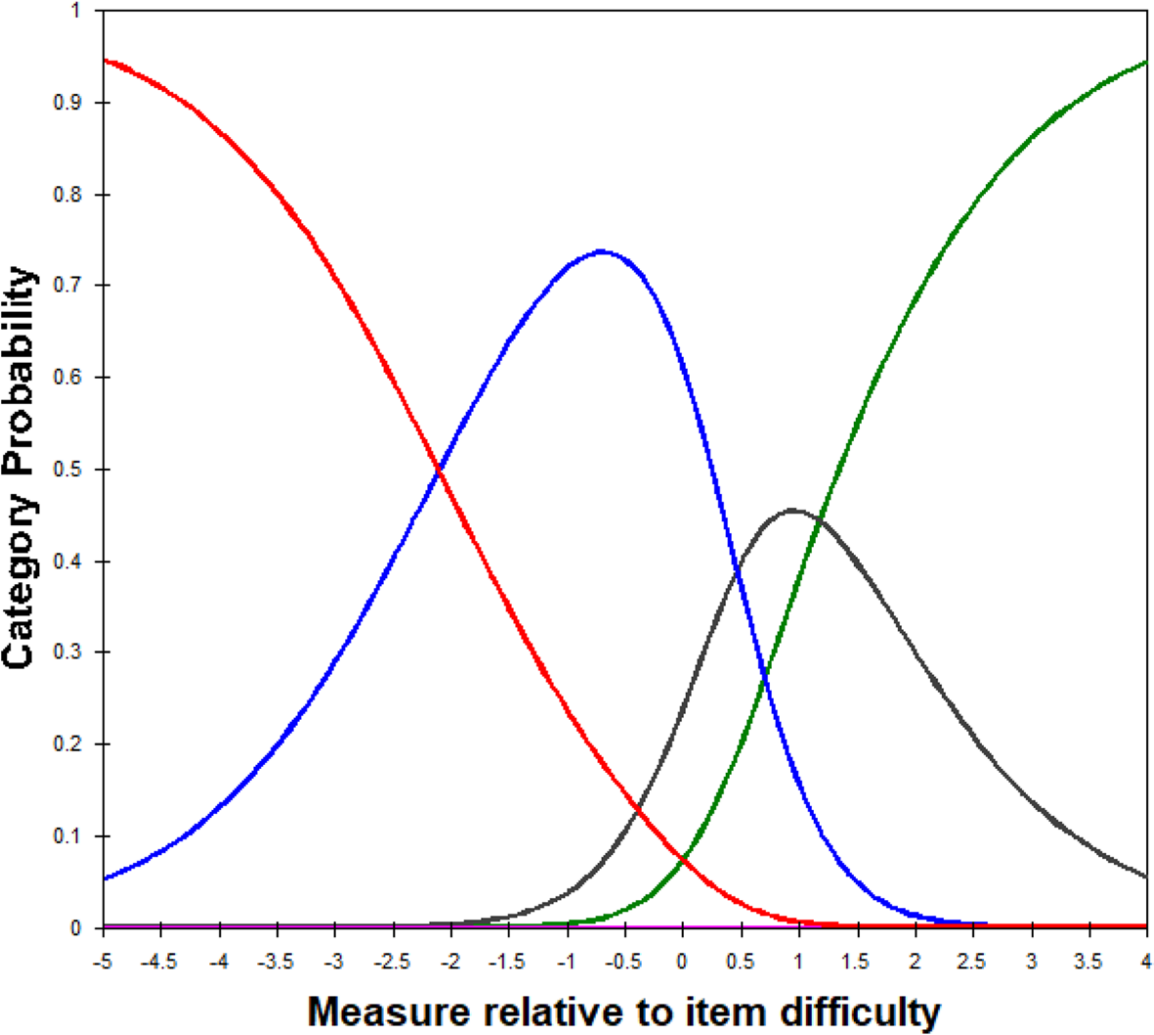
Category probability curve for the functional scale of the Final Malay QIRC. Red = Category 1 (*not at all/never*); Blue = Category 2 (*little/occasionally*); Black = Category 3*,* (*a lot/very often*); Green = Category 4 (*always/extremely*)

The category probability curve illustrates that the modified four response categories for the functional scale were ordered (Fig. 2). The original five response categories for the emotional scale were also ordered.

After modifying the items and the response categories, the higher person reliability and separation index of the 12-item functional and the 7-item emotional scales were found. These person separation indices demonstrated that the 19-item Final Malay QIRC could secede the participants into distinct strata following their ability. The item reliability and separation index also showed that participants were able to differentiate items according to their difficulty hierarchy (Table 2).

**Table 2:** Rasch analysis outcomes of person-item parameters for the Malay QIRC

	Malay QIRC			
	20-item Pilot (***N*** = 105)		19-item Final (***N*** = 304)	
Parameters	Functional	Emotional	Functional	Emotional
Item				
Total	13	7	12	7
Removed	–	–	Item 3	–
Number	1–13	14–20	1–12	13–19
Category				
Total	5	5	4	5
Collapsed	–	–	Category 3	–
Ordered?	No	Yes	Yes	Yes
Person reliability	0.80	0.79	0.80	0.81
Person separation index	1.98	1.93	2.01	2.06
Item reliability	0.98	0.93	0.99	0.98
Item separation index	6.62	3.77	10.45	6.45
Average infit, MnSq (z-std)				
Participant ability	1.02 (0.0)	0.98 (−0.3)	0.99 (− 0.1)	0.96 (− 0.2)
Item difficulty	1.00 (0.0)	1.01 (0.0)	0.99 (− 0.2)	1.02 (0.1)
Average outfit, MnSq (z-std)				
Participant ability	1.01 (0.0)	0.98 (−0.3)	1.00 (0.0)	0.97 (−0.2)
Item difficulty	1.01 (0.0)	0.98 (−0.1)	1.01 (− 0.1)	0.97 (− 0.4)

*QIRC* Quality of Life Impact Refractive Correction, *N* number of participants, *MnSq* mean square, *z-std* z-standardised

The fit statistics exposed that the average infit and outfit of the person-item on both the functional and emotional scales were approximately 1.0 MnSq (Table 2). Furthermore, the infit and outfit for all items were within 0.66 to 1.45 MnSq (Table 3). Overall, the 19-item Final Malay QIRC showed a satisfactory fit to the Rasch model.

**Table 3:** Item calibration, infit and outfit for the Final Malay QIRC

Item no.	Item description	Item calibration^**a**^ (SE)	Infit MnSq	Outfit MnSq
1	How much difficulty do you have driving in glare conditions?	1.00 (0.07)	1.24	1.45
2	During the past month, how often have you experienced your eyes feeling tired or strained?	1.23 (0.05)	0.88	0.88
3	How much trouble is having to think about your spectacles/contact lenses/refractive surgery, e.g. LASIK, before doing things; (e.g. travelling, sport, going swimming)?	0.30 (0.07)	1.09	1.12
4	How much trouble is not being able to see when you wake up; (e.g. to go to the bathroom, look after a baby, see alarm clock)?	0.18 (0.07)	1.11	1.08
5	How much trouble is not being able to see when you are on the beach or swimming at the beach or pool, because you do these activities without spectacles or contact lenses?	− 0.58 (0.07)	1.01	0.89
6	How much trouble is your spectacles or contact lenses when you wear them during exercises or sports activities?	0.74 (0.07)	1.03	1.02
7	How concerned are you about the initial and ongoing cost to buy your current spectacles/contact lenses/refractive surgery, e.g. LASIK?	−0.17 (0.08)	0.84	0.80
8	How concerned are you about the cost of unscheduled maintenance of your spectacles/contact lenses/refractive surgery, e.g. LASIK; (e.g. breakage, loss, new eye problems)?	−0.35 (0.08)	0.91	0.89
9	How concerned are you about having to increasingly rely on your spectacles or contact lenses since you started to wear them?	−0.75 (0.06)	1.02	1.06
10	How concerned are you about your vision not being as good as it could be?	−0.70 (0.06)	0.72	0.66
11	How concerned are you about medical complications from your choice of optical correction (spectacles, contact lenses and/or refractive surgery, e.g. LASIK)?	−0.59 (0.08)	0.88	0.87
12	How concerned are you about eye protection from ultraviolet (UV) radiation?	−0.32 (0.07)	1.21	1.33
13	During the past month, how much of the time have you felt that you have looked your best?	−0.02 (0.07)	1.05	1.02
14	During the past month, how much of the time have you felt that you think others see you the way you would like them to (e.g. intelligent, sophisticated, successful, cool)?	0.47 (0.07)	1.15	1.10
15	During the past month, how much of the time have you felt complimented/flattered?	1.05 (0.06)	1.20	1.08
16	During the past month, how much of the time have you felt confident?	−0.08 (0.07)	0.82	0.80
17	During the past month, how much of the time have you felt happy?	−0.81 (0.05)	0.80	0.81
18	During the past month, how much of the time have you felt able to do the things you want to do?	−0.35 (0.06)	1.00	0.97
19	During the past month, how much of the time have you felt eager to try new things?	−0.26 (0.07)	1.09	1.02

Items 1 to12 are functional scale; Items 13 to19 are emotional scale

*no.* number, *SE* standard error, *MnSq* mean square, *LASIK* laser-assisted in situ keratomileusis

^a^Item calibration in logit unit

**Fig. 3:**
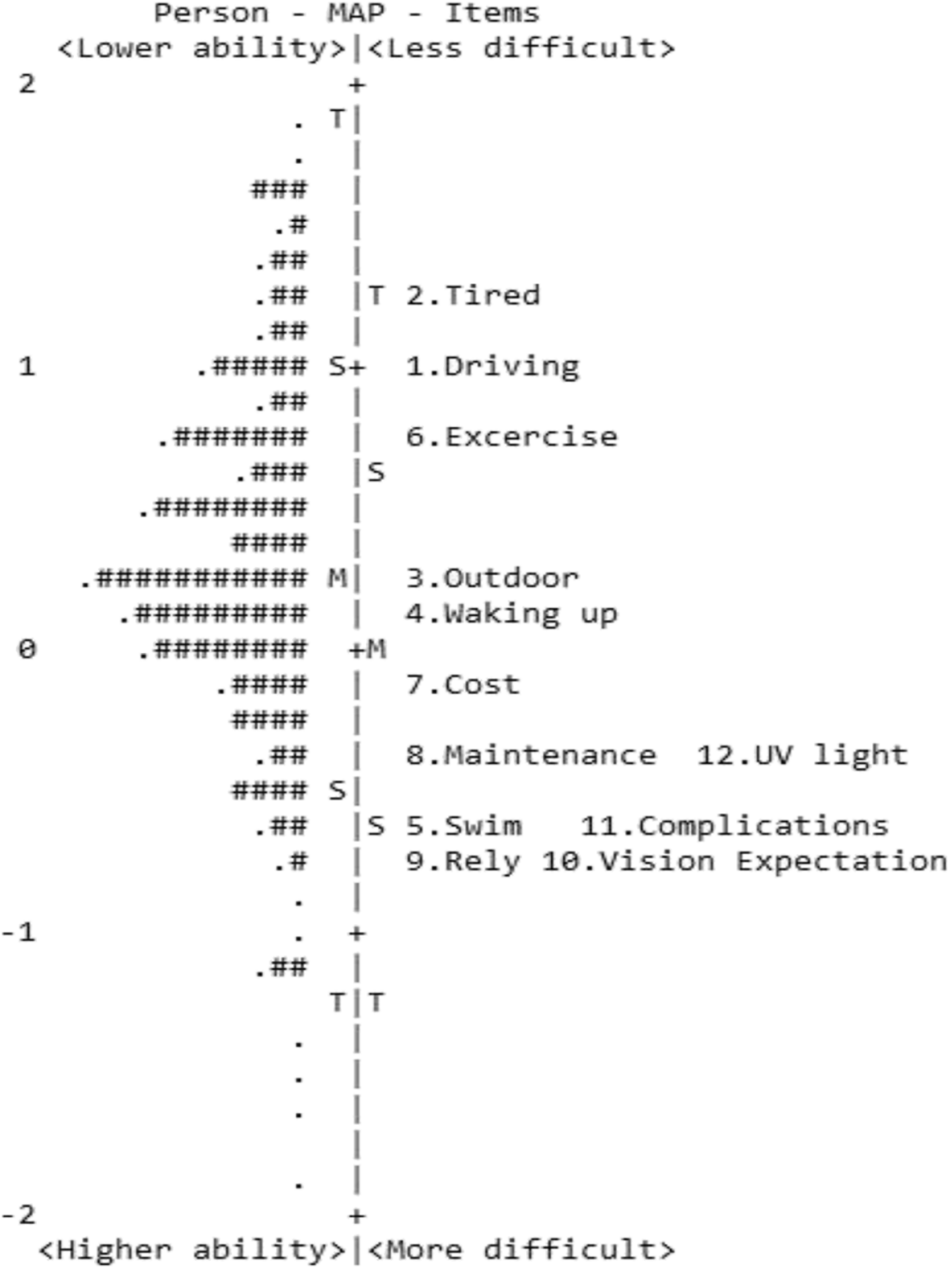
Person-item map for the functional scale of the Final Malay QIRC. The left side of the dashed line represents the participants, with lower ability participants near the top of the map. The right side of the dashed line represents the items, with less difficult items near the top of the map. Each ‘*#*’ = three participants; each ‘.’ = one participant; M = mean; S = one standard deviation; T = two standard deviations

**Fig. 4:**
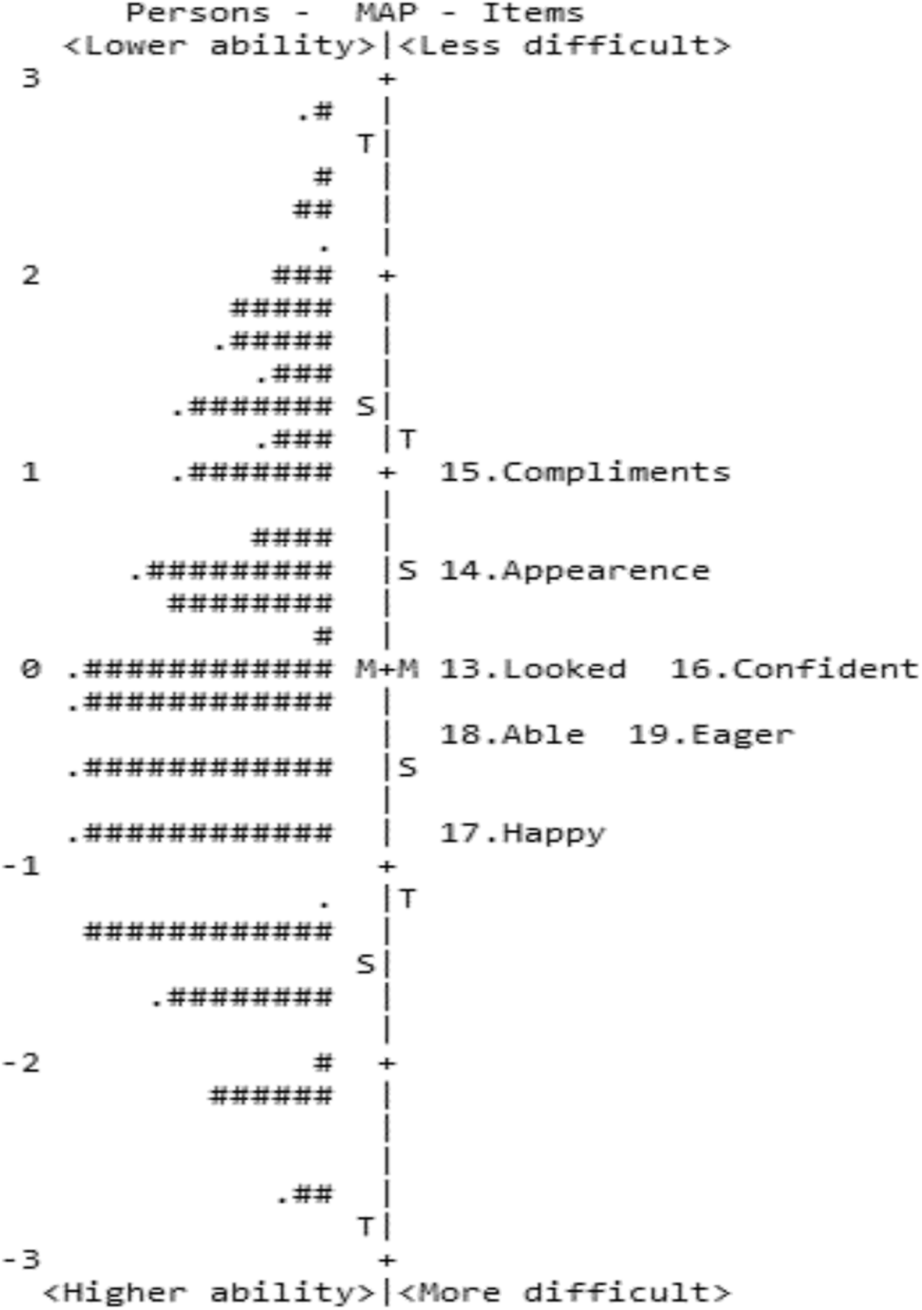
Person-item map for the emotional scale for the Final Malay QIRC. The left side of the dashed line represents the participants, with lower ability participants near the top of the map. The right side of the dashed line represents the items, with less difficult items near the top of the map. Each ‘*#*’ = three participants; each ‘.’ = one participant; M = mean; S = one standard deviation; T = two standard deviations

Targeting precision assessment was illustrated by the item difficulty and participant ability distribution and their mean difference in the person-item map (Figs. 3 and 4). The mean difference (targeting) on the functional and emotional scales was 0.23 and 0.08 logits, respectively. The differences were considerably small. On the functional scale, Items 2 and 1 (in sequential order) were the two least difficult items, whereas Items 9 and 10 were the most difficult for participants to answer (Fig. 3). On the emotional scale, Item 15 was the least difficult item, while Item 17 was the most difficult item (Fig. 4).

Analysis of the DIF revealed that all items of the functional and emotional scales had a DIF contrast of less than 0.50 logits. However, Item 18 ‘*able to do the things you want to do’* showed a significant probability between genders (DIF contrast, 0.40 logits; *t* = 2.12, *p* = 0.04). It indicated that Item 18 was 0.40 logits tougher for females than for males.

There was no significant difference in QIRC scores between the test and retest (mean difference, 1.09 ± 4.07 units; *t =* 2.05, *p* = 0.05). Test-retest reliability analysis showed a high ICC (single measures, 0.94) and Cronbach’s α (0.97) with a CoR of ±8.14 units. In addition, the time required to complete the questionnaire between the first and second visits was insignificant (mean difference, 0.12 ± 0.70 min; *t* = 1.33, *p* = 0.19). These findings confirmed that the 19-item Final Malay QIRC had excellent repeatability and internal consistency.

## Discussion

The Malay-translated version of the QIRC was subjected to a proper validation process using Rasch analysis and test-retest reliability analysis. The validity and reliability analysis demonstrated that the Final Malay QIRC was well-targeted, reliable, internally consistent, and had ordered response categories.

Rasch analysis revealed that the Malay QIRC scale was multidimensional in assessing the QoL of spectacle and contact lens wearers. This finding is in line with those found in existing works, where Ang et al. [34] analysed the original QIRC, and Kaphle et al. [9] validated the Chichewa QIRC, reporting similar observations. A review article also noted that QoL instruments are often described as multidimensional [35]. Hence, analysis of the Malay QIRC was split into the functional (e.g., ‘*difficulty driving in glare conditions*’ and ‘*experiencing eye strain*’) and emotional scales (e.g., ‘*looked best*’ and ‘*felt complimented*’) to obtain an accurate result [34]. This suggests that Rasch analysis should definitely be used when validating an instrument for a specific population.

The modified response categories for the functional scale performed well to differentiate participants’ categories. Participants could use the categories to classify the four difficulty levels of the items [20]. The original response categories for the emotional scale also worked well. These category thresholds proved that both response categories functioned appropriately for the Final Malay QIRC.

The person reliability and separation index indicated that the 19-item Final Malay QIRC could discriminate the participants into distinct strata, from poor to excellent QoL. Removal of one misfit item and cross-cultural adaptation of several items improved the person reliability and separation index on both the functional and emotional scales. The item reliability and separation index attained a good separation level for participants to rank the item difficulty levels. This conveyed that the number of participants recruited in this study was adequate for the validation process [28].

Linacre [28] outlined that an individual item with an infit and outfit outside the range of 0.5 to 1.5 MnSq or equal to/greater than 2.0 z-std is considered a misfit item. Hence, the researchers decided to omit Item 3 ‘*difficulty is not being able to use non-prescription* s*unglasses’* [36] from the Malay QIRC. Approximately 40% of the participants in the pre-test rated Item 3 as ‘*not applicable’* or *‘none’*. The researchers postulated that photochromic lenses were commonly used as an alternative to shade the eyes among spectacle wearers in this cohort.

The person-item maps depict that item difficulty and participant ability were evenly distributed, designating that the difficulty range of the final 19-item matched to the ability continuum of the 304 participants [37]. Moreover, the mean differences were even smaller than 0.29 logits, indicating good targeting. These demonstrate that the 19-item Final Malay QIRC were sufficiently targeted to individuals with optical corrections [29].

On the functional scale, the items *‘difficulty driving in glare conditions’* and *‘experiencing eye strain’* were the two easiest items to answer, representing the most related items to QoL of spectacle and contact lens wearers in Malaysia. These findings are echoed with a comparative study on QIRC scores between refractive correction groups [12]. The previous study reported that the spectacle and contact lens groups had lower QIRC scores on the same items than the refractive surgery group [12]. In contrast, the Chichewa QIRC study found that *‘concern about the initial and ongoing cost to buy spectacles/contact lenses’* was the easiest item, reflecting the greatest relationship to the Malawian population [9]*.* Malawi is a low-income country with a limited-resource setting [9], while Malaysia is a developing country that has an accessible healthcare system, including optical correction resources.

On the emotional scale, the easiest item was the question about *‘felt complimented’,* and the most difficult was the question for *‘felt happy*’*.* Unlike the Malawi study, the most difficult emotional item was the question ‘*felt eager to try new things*’ [9]*.* This is most likely attributable to the cultural and environmental differences in the population studied.

A noticeable DIF was found between males and females in Item 18 ‘*able to do the things you want to do’*. Nevertheless, the DIF contrast was not greater than 0.50 logits. The variability in item difficulty between genders could be due to the different preferred activities and hobbies of males and females. Concerning the DIF contrast was still lower than 0.50 logits, no amendment has been made to Item 18. In order to understand why participants responded differently to the item and whether DIF had a major effect on the overall score, further research should be carried out [38].

The test-retest results found that the 19-item Final Malay QIRC had good repeatability like the original QIRC [7]. Moreover, Cronbach’s α indicated that the 19-item possessed high internal consistency [39]. Therefore, it can be concluded that the Malay QIRC version is a reliable instrument for assessing the QoL of people with refractive correction.

The unequal number of participants with spectacle and contact lens correction is a notable limitation of this study. However, the item separation indices showed that the number of participants was sufficient to distinguish the item difficulty order. Another possible limitation is that no refractive surgery participant was recruited to validate the Malay QIRC. Hence, the Malay version of the QIRC might not be applicable to the refractive surgery group, whereby another validation study is warranted.

## Conclusions

In conclusion, the Malay-translated version of the QIRC has good psychometric characteristics to evaluate the QoL of refractive correction wearers in Malaysia. The Malay QIRC was appropriately translated, cross-culturally adapted for the Malaysian population, and properly validated using Rasch analysis. Rasch analysis suggested that the functional and emotional Malay QIRC scales should be split for analysis. This valid and reliable questionnaire may be practical to be administered in routine ophthalmic practice, and the QoL outcomes may serve as a guide for better refractive management.

## Data Availability

The datasets used and/or analysed during the current study available from the corresponding author on reasonable request.

## Abbreviations

CIOMS: Council for International Organisations of Medical Sciences
CoR: Coefficient of repeatability
D: Dioptre
DIF: Differential item functioning
ICC: Intraclass coefficient
ICH-GCP: International Conference of Harmonisation Good Clinical Practice Guideline
MnSq: Mean square
NAVQ: Near Activity Visual Questionnaire
PCAR: Principal components analysis of residuals
QIRC: Quality of Life Impact on Refractive Correction
QoL: Quality of life
QoV: Quality of Vision
SD: Standard deviation
SER: Spherical equivalent refraction
SPSS: Statistical Package for the Social Sciences
z-std: z-standardised
*α*: Alpha
